# Beneficial Effect of Kidney Bean Resistant Starch on Hyperlipidemia—Induced Acute Pancreatitis and Related Intestinal Barrier Damage in Rats

**DOI:** 10.3390/molecules27092783

**Published:** 2022-04-27

**Authors:** Zhaohang Zuo, Shuting Liu, Weiqiao Pang, Baoxin Lu, Wei Sun, Naidan Zhang, Xinyu Zhou, Dongjie Zhang, Ying Wang

**Affiliations:** 1 College of Food Science, Heilongjiang Bayi Agricultural University, Daqing 163319, China; byndzzh1994@163.com (Z.Z.); xkljcg@163.com (S.L.); hljbypwq@163.com (W.P.); bynd2020ztgzy@163.com (B.L.); swsw0102@163.com (W.S.); zndan1025@163.com (N.Z.); zxy199810271015@163.com (X.Z.); 2 National Coarse Cereals Engineering Research Center, Daqing 163319, China

**Keywords:** kidney bean resistant starch, hyperlipidemia, acute pancreatitis, intestinal barrier damage

## Abstract

Accumulating attention has been focused on resistant starch (RS) due to its blood-lipid-lowering activities. However, reports on the potential bioactivities of RS for preventing hyperlipidemia acute pancreatitis (HLAP) are limited. Therefore, in this study, an acute pancreatitis model was set up by feeding a hyperlipidemia diet to rats, and subsequently evaluating the anti-HLAP effect of RS in kidney beans. The results show that the IL-6, IL-1β, and TNF-α of serum in each RS group were decreased by 18.67–50.00%, 7.92–22.89%, and 8.06–34.04%, respectively, compared with the model group (MOD). In addition, the mRNA expression of tight junction protein ZO-1, occludin, and antibacterial peptides CRAMP and DEFB1 of rats in each RS group increased by 26.43–60.07%, 229.98–279.90%, 75.80–111.20%, and 77.86–109.07%, respectively. The height of the villi in the small intestine and the thickness of the muscle layer of rats were also increased, while the depth of the crypt decreased. The present study indicates that RS relieves intestinal inflammation, inhibits oxidative stress, and prevents related intestinal barrier damage. These results support the supplementation of RS as an effective nutritional intervention for HLAP and associated intestinal injury.

## 1. Introduction

Acute pancreatitis (AP) is a serious comprehensive digestive system disease caused by a variety of exogenous or endogenous factors that cause the activation and release of pancreatic acinar trypsin, which induces pancreatic autophagy and digestion, thereby triggering local pancreatic inflammation and systemic organ failure [1]. Clinical investigations have found that about 34% of patients with acute pancreatitis also suffer from dyslipidemia, indicating that hyperlipidemia has gradually become a common etiology of hyperlipidemia acute pancreatitis (HLAP) [2,3]. During the early phase of HLAP, trypsinogen produced in situ in the pancreas is abnormally activated, leading to pancreatic tissue self-destruction. This injury induces a strong systemic inflammatory response released into circulation by pro-inflammatory molecules (such as interleukins, tumor necrosis factor, and platelet activation factor) [4]. The above inflammatory factors can damage the intestinal mucosal barrier and cause intestinal dysfunction, which is the inevitable result of the development of acute pancreatitis. Therefore, effective control of intestinal tissue structure and function damage can delay the development of HLAP [5,6]. On another side, the triglyceride (TG), total cholesterol (TC), and low-density lipoprotein cholesterol (LDL-C) in the lipid profile of HLAP were significantly increased (*p* < 0.05). HLAP is related to lipid metabolism [7].

In line with the side effects of statins and the intense pain patients suffer, there is accumulating interest in using dietary botanical supplements for prevention and adjuvant therapy. Studies on dietary factors and blood lipid levels have shown that different dietary patterns impact blood lipid levels to a certain extent, and a plant-based diet is a protective factor for dyslipidemia [8]. The compact molecular structure of RS can hinder the digestion of various enzymes [9]. RS improves gastrointestinal health, affects the composition of the intestinal flora, potentially affects blood lipid metabolism, and inhibits inflammatory cytokines by producing gas (methane, hydrogen, carbon dioxide) and short-chain fatty acids (formic acid, acetic acid, propionic acid, etc.) during colonic fermentation [10]. In addition, RS can regulate glucose metabolism by down-regulating the expression level of key enzymes, and also restore normal levels of insulin by repairing damaged pancreatic β cells [11]. After ingesting RS, the concentration of HDL-C in a hamster study increased, and the concentration of TG, TC, and LDL-C reduced, repairing the damage to the cecum and colon tissue caused by a high-fat diet [12]. RS supplementation has obvious effects on lowering TC and LDL-C, and a longer time (>4 weeks) of supplementation can provide a more robust effect [13]. The metabolic effects of RS3 on T2DM have been studied, indicating that RS3 can down-regulate the levels of blood glucose, improve dyslipidemia, reduce insulin resistance, and enhance insulin sensitivity [14]. GB (rich in resistant starch) in the diet of high-fat-fed mice increased SCFA production, down-regulated the expression of genes involved in lipogenesis, and enhanced the expression of transport proteins involved in lipid excretion [15]. The rice starch-FA complex (resistant starch V) can reduce the bodyweight of rats under a high-fat diet, and improve serum lipid profiles, oxidative stress, and liver function [16]. In summary, RS may be a promising food in diet therapy for obesity, hyperlipidemia, and T2DM. However, the effect of RS on HLAP is still unknown.

In view of these facts, we explored whether kidney bean RS and its metabolites can alleviate HLAP in rats and restore the intestinal barrier damage caused by inflammatory cytokines.

## 2. Results

### 2.1. Molecular Structure and Digestibility of RS

**Figure 1:**
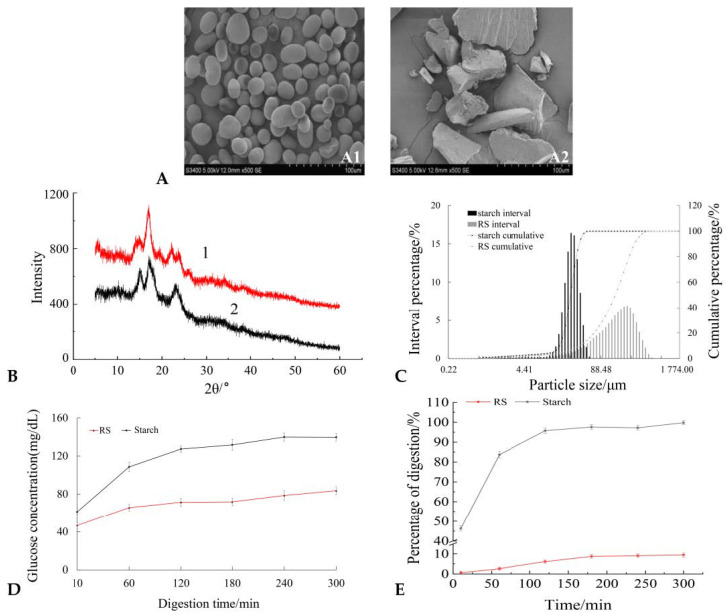
Molecular structure and digestibility of RS. (**A**) Scanning diagram of the morphology of kidney bean starch (A1) and RS (A2); (**B**) X-ray diffraction spectra of kidney bean RS (1) and starch (2); (**C**) size distribution of kidney bean RS and starch; (**D**) digestive characteristics of kidney bean RS and starch; (**E**) digestion ratio of kidney bean RS and starch at different times.

Comparing purified RS with starch, it was found that kidney bean RS granules have an irregular and angular polygonal structure with a compact texture, rough surface, and a lamellar structure on the cross-section (Figure 1A). X-ray diffraction results show that RS based on a renal bean starch crystal structure had new diffraction peaks at 6°, 22°, and 24°, and the new diffraction peak was the characteristic diffraction peak of B-type crystals, so RS exhibited a combination of A and B to form a C-type crystal structure (Figure 1B). Compared with the original starch, the volume average particle size of the RS granules increased significantly, and the specific surface reduced significantly. The reduction in a specific surface area can avoid excessive contact between RS and enzymes, thereby enhancing its resistance to enzymatic hydrolysis (Table 1, Figure 1C). The above results show that the internal structure of RS is stable from the perspective of micro-morphology, crystal structure, molecular mass, and particle size.

Subsequently, we applied RS and starch to obtain the vitro digestion ratio (Figure 1D,E). The digestion process of the sample involved three steps to simulate oral chewing in the mouth, digestion by the stomach, and the small intestine in vitro digestion [17]. We were intuitively exploring the final digestion rate of carbohydrates through releasing sugars in the digestive juice of the small intestine. The results show that the glucose concentration in the RS digestive fluid was significantly lower than that of starch at the same digestion time. At the same time, the digestibility of resistant starch was much lower than that of native starch in the same period. Combined with the previous structural properties of resistant starch, it is known that resistant starch has good resistance to enzymatic hydrolysis in the digestive system, which may be beneficial to its physiological function in the intestine [18].

### 2.2. Dietary Intake Affected the Body Weight and Pancreas Weight of HLAP Rats

The body weight and pancreas mass of the rats were measured, and the trend of body weight and related organ index were monitored. The results show that the rats in the CON had a slower weight increase trend than those in the other groups because they were fed with a common diet. After oral administration of RS and simvastatin, compared with the MOD, the rats in the SV and the RS groups grew slowly or even slightly, indicating that the RS can control the weight to a normal extent (Table 2). The pancreas coefficient of the MOD was significantly different from that of the CON (*p* < 0.05), indicating that intraperitoneal injection caused damage to the rat pancreas, and the pancreas index decreased, indicating that the pancreas may suffer from atrophy or degenerative disease. After treatment with positive drugs and RS, the pancreas index of each RS group of rats was 12.46%, 34.26%, and 44.64% higher than that in the MOD.

### 2.3. The Effect of RS on Serum Pathological Parameters in Rats

Serum triglyceride, amylase, and lipase concentrations are common indicators of clinical diagnosis of acute pancreatitis. The blood lipid and serum levels of AMLY and LIPA in the rats are shown in Table 3. After being fed a high-fat diet and given intraperitoneal injection of meninges, the contents of TC, TG, and AMLY and LIPA in the serum of the MOD increased significantly (*p* < 0.05) compared with the CON, indicating that the HLAP model was successfully induced. After RS and positive drug intervention, compared with the MOD, the blood lipid and serum levels of AMLY and LIPA in the rats in each RS group increased by 16.68–31.05% and 21.97–48.55%, respectively. Moreover, the TC and TG content in each RS group increased significantly by 43.14–69.36% and 12.61–43.69% compared to that in the MOD (*p* < 0.05). All of the improvements in the H-RS were significantly better than those in the L-RS.

### 2.4. Intaking of RS Affects the Degree of Edema and Myeloperoxidase Activity in Pancreatic Tissue of Rats in Each Group

**Figure 2:**
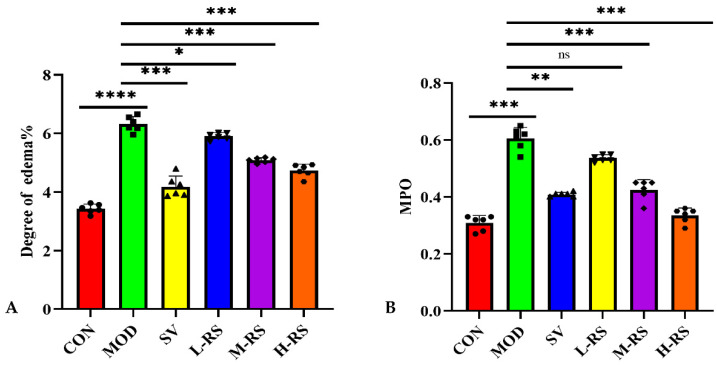
The degree of pancreatic edema on rats (**A**) and MPO activity in pancreatic tissues of rats (**B**) in each group (ns *p* > 0.05; * *p* < 0.05; ** *p* < 0.01; *** *p* < 0.001; **** *p* < 0.0001 versus MOD, and the points of different shapes represent the actual value of each sample, *n* = 6).

The degree of pancreatic organ edema could directly reflect the severity of pancreatic injury and HLAP in rats. As shown in Figure 2A, the wet/dry mass ratio of the pancreas in the MOD was significantly higher than that in the CON, indicating that the MOD had pancreatic damage and obvious edema. Compared with the MOD, the degree of pancreatic edema of H-RS and M-RS decreased to varying degrees (25.24% and 19.46%), after the intervention of RS. However, they are less effective than SV (33.84%). Myeloperoxidase (MPO), as a heme protease, mainly exists in neutrophils and monocytes, and the activity change is the key symbol of neutrophil function and activation. As shown in Figure 2B, the MPO activity of pancreatic tissue in the MOD was significantly higher than that in the CON (*p* < 0.001), indicating that the inflammatory cells in the MOD were severely aggregated and infiltrated. After gavage treatment and positive drug intervention, compared with the MOD, the MPO activity of each RS group decreased by 11.53–44.81%. The inhibitory effect of the H-RS and M-RS on MPO activity was significantly better than that of L-RS, and even the H-RS had a better effect than SV.

### 2.5. The Results of the Pancreas Pathology Section

**Figure 3:**
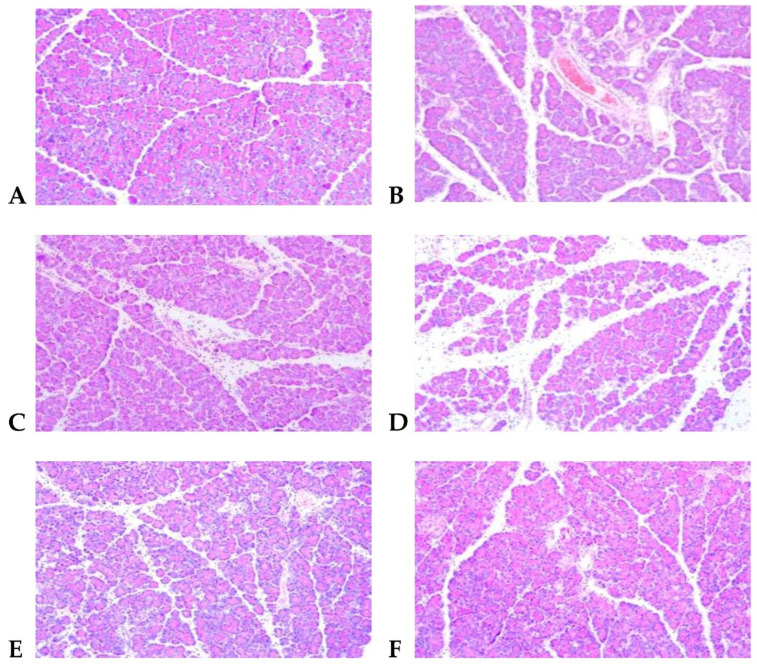
HE staining observation of rat pancreas pathology (×100). (**A**) CON; (**B**) MOD; (**C**) SV; (**D**) L-RS; (**E**) M-RS; (**F**) H-RS.

The pancreas tissue of rats in the CON was in the normal range (Figure 3). The acetic acid original was intact and uniformly distributed. The lobular veins were clear, and there was no cell necrosis or inflammatory cell infiltration. Conversely, the pancreatic lobular space of the MOD was significantly wider than that of the CON, and a large number of monocytes and neutrophils infiltrated around the necrotic focus, which was consistent with the symptoms of edema-type acute pancreatitis. In the SV, after oral administration of simvastatin and intravenous injection of simvastatin, the pancreatic tissue structure was relatively clear, acinar vacuoles and swelling and necrosis were reduced, and inflammatory cell infiltration was relieved. After gavage of RS, compared with MOD rats, pancreatic tissue damage reduced to varying degrees in rats in the RS groups; pancreas tissue structure was more complete in M-RS and H-RS, with dense lobular stroma, as well as acinar cell necrosis and congestion. In addition, inflammatory cell infiltration reduced significantly; in the L-RS, the pancreas tissue of the rats still showed lobular interstitial looseness and focal acinar cell necrosis, which revealed that high-dose RS could effectively alleviate acute edema-induced pancreatic damage in rats induced by the peritoneum.

### 2.6. Effect of Kidney Bean RS on Secretion of Inflammatory Cells in Rats

The onset of acute pancreatitis and hyperlipidemia induces pancreatic acinar cells to release pro-inflammatory cytokines, such as IL-6, IL-1β, TNF-α, etc., through activating the nuclear transcription factor NF-κB to produce a large number of inflammatory mediators, which trigger multiple organ dysfunction or systemic inflammatory response syndrome to aggravate the condition. Therefore, the course of HLAP and the degree of inflammation development can be analyzed according to the concentration of inflammatory factors. The inflammatory cell secretion level of the rats in each group was measured, and the result is shown in Table 3. The serum level inflammatory factors of rats in the MOD were significantly higher than those in the CON (*p* < 0.05). After the intervention of positive drugs and RS, compared with the MOD, the serum levels of IL-6, IL-1β, and TNF-α in each RS group reduced by 18.67–50.00%, 7.92–22.89%, and 8.06–34.04%, respectively. The regulatory effect of RS on the inflammatory factors was positively correlated with the dose; especially, H-RS could effectively inhibit the release of inflammatory factors.

### 2.7. Effects of Kidney Bean RS on Serum DAO, DLA, ET, and sIgA Levels in Rats

The levels of serum DAO, DLA, and ET in the MOD were higher than those in the CON, while the levels of sIgA decreased (*p* < 0.05) (Table 3). After gavage treatment, the serum DAO, DLA, and ET levels of rats in each RS group, respectively, decreased to varying degrees (9.56–24.13%, 5.16–26.70%, and 5.71–23.66%) compared with the MOD. The levels of sIgA increased by 2.52–19.25%. Except for the DLA in L-RS, the levels of DAO, DLA, ET, and sIgA in the serum were significantly different from the MOD (*p* < 0.05).

### 2.8. Effect of Kidney Bean RS on Histopathological Sections of Rat Small Intestinal Mucosa

**Figure 4:**
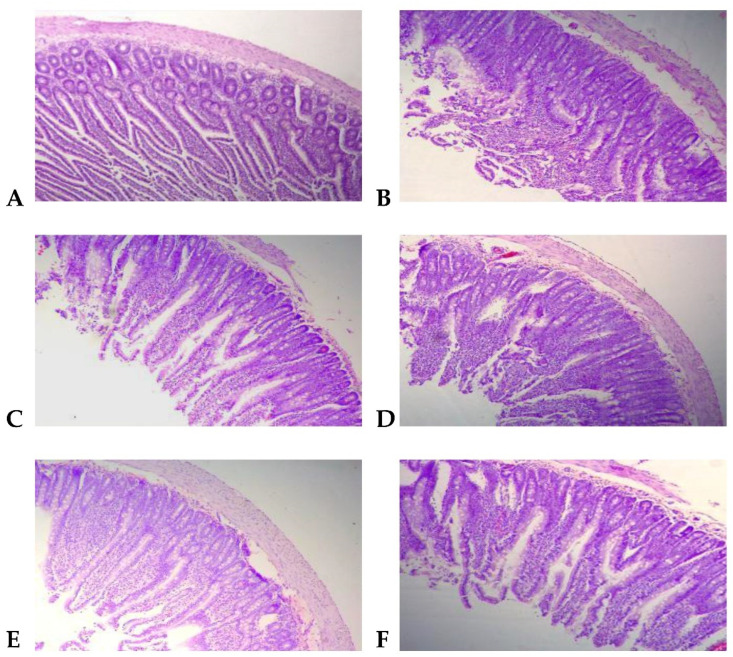
HE staining of the small intestine in each group of rats (×100). (**A**) CON; (**B**) MOD; (**C**) SV; (**D**) L-RS; (**E**) M-RS; (**F**) H-RS.

In the MOD, the intestinal mucosa was damaged, and the intestinal villi were lodging and falling off. The height of the intestinal villi was reduced and uneven. The thickness of the intestinal mucosa muscle layer reduced, the gap was wide, and the crypt depth increased. After oral administration, the intestinal morphology of the rats in each group was relatively complete and hierarchical. Compared with the MOD, the intestinal mucosa infiltration was seen in the L-RS, and the inflammatory cell infiltration in the M-RS and H-RS was relatively less. The height of the villi in the small intestine and the thickness of the muscle layer of rats increased, and the depth of the crypt decreased, in each RS group. The intestinal mucosa was tightly connected, and the columnar epithelial cells were relatively complete. It was shown that kidney bean RS could prevent small intestinal mucosal mechanical barrier damage in HLAP rats to a certain extent (Figure 4).

### 2.9. Intestinal Functional Protein mRNA Level and Protein Expression

**Figure 5:**
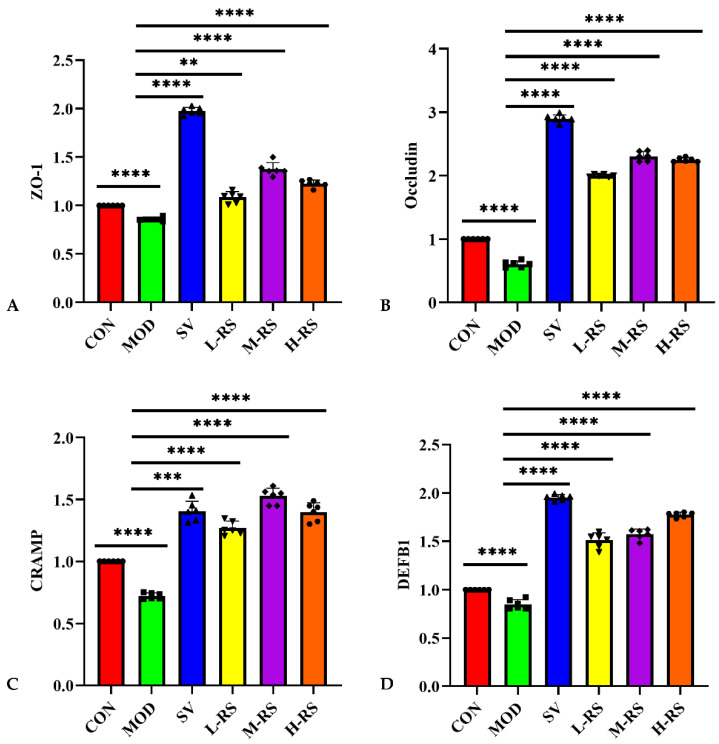
Effect of kidney bean RS on ZO-1 (**A**), occludin (**B**), CRAMP (**C**), and DEFB1 (**D**) gene expression in rat intestine (** *p* < 0.01; *** *p* < 0.001; **** *p* < 0.0001 versus MOD, and the points of different shapes represent the actual value of each sample, *n* = 6).

Gene expression levels of rats in the CON were set as controls, and mRNA expression levels of ZO-1, occludin, CRAMP, and DEFB1 in the MOD rats reduced significantly (*p* < 0.0001). The RS was gavaged before caerulein induced HLAP. Compared with the MOD, the mRNA expression levels of tight junction protein ZO-1, occludin, and antibacterial peptides CRAMP and DEFB1 of rats in each RS group increased by 26.43–60.07%, 229.98–279.90%, 75.80–111.20%, and 77.86–109.07%, respectively (Figure 5).

## 3. Discussion

This study explored the protective effect of RS on acute pancreatitis and related intestinal barrier damage in rats with hyperlipidemia. The core index results of acute pancreatitis in each group of rats confirmed that HLAP raised the serum AMLY and LIPA levels to 3 times higher than normal levels. Meanwhile, it damaged the pancreatic tissues and led to edema of the pancreatic organs. However, dietary supplementation of RS effectively reduced the levels of AMLY and LIPA in the serum of rats among groups, reduced the degree of pancreatic edema, and inhibited the activity of MPO in pancreatic tissue. Combined with pathological sections, the pancreas of the rats in the RS groups had no obvious lipid vacuoles, and the inflammatory infiltration was reduced, which further proved that the intake of RS can effectively protect the pancreas from damage. Researchers have found that dietary fiber intake correlates with pancreatic enzyme activity. Dietary fiber can inhibit trypsinogen digestive activity through a certain pathway, preventing pancreatic tissue damage [19,20]. At present, some relevant studies have proved that HLAP is a fatal disease that can lead to organ failure [21,22]. Its premature activation of pancreatic proteases in pancreatic acinar cells causes the process of self-digestion of the pancreas parallel to the immune response, and hyperlipidemia aggravates the immune response, which promotes the strength of the immune response to determine systemic complications and disease severity [23]. Therefore, we explored the protective effect of RS on HLAP rats and intestinal barrier damage from the following two aspects: (1) its regulation of blood lipid levels; (2) its secretion of inflammatory cytokines during the course of HLAP inhibition.

As the acinar body is damaged during its own digestion process, it excessively stimulates pancreatic parenchymal inflammation, which implies that the pathogenesis of HLAP-induced neutrophils and macrophages is infiltrated by inflammatory mediators to release inflammatory markers such as IL-6, IL-1β, TNF-α, etc. These factors activate NF-κB and then produce more inflammatory mediators, causing more serious MODS and SIRS [24,25]. Among them, TNF-α is the first pro-inflammatory cytokine released in the course of HLAP, which could stimulate the secretion of IL-6 and IL-1β. IL-6 and IL-1β are genera of ILs, which promote the development and differentiation of specific lymphocytes and activate inflammatory mediators, which in turn stimulate other immune cells to secrete TNF-α, and the three interact with each other in a vicious cycle, causing severe damage to the pancreas and related organ tissues [26,27]. The results show that RS reduces the inflammatory cytokine cycle and has a certain immune regulating effect. Nilsson et al. found that consumption of RS by the body increased glucagon-like peptide (GLP-2), affected the activity of intestinal hormones, and thus suppressed the increase in inflammatory markers [28]. In this experiment, the levels of serum TNF-α, IL-6, and IL-1β in rats in each RS group after gavage were lower than those in the MOD, especially in the M-RS and H-RS, which were significantly reduced. It was proved that the intake of RS could effectively inhibit the inflammatory response of HLAP rats, and that the medium and high doses of RS had a stronger inhibitory effect on the secretion of inflammatory cytokines.

From a pathological point of view, researchers generally believe that HLAP is caused by too many free fatty acids decomposed by triglycerides, which disrupt many acinar functions like exocytosis enzyme activation [29,30]. It causes premature activation of trypsinogen and self-digestion, thereby damaging pancreatic tissue. Zhang found that TG levels are directly related to the severity of AP disease. In the onset of AP, rapidly reducing TG levels can block the progress of SIRS in time [31]. The results of this experiment confirm that the body weight and blood lipid concentration of rats in the RS groups were significantly lower than in the MOD. Related studies have demonstrated that dietary intake of RS reduces the levels of serum TC and TG by stimulating the secretion of bile acids and promoting cholesterol and lipid excretion, and stimulates the synthesis of gastrointestinal peptide hormones to avoid diet-induced obesity and hyperlipidemia [32,33].

In addition, the intestinal barrier serves as an effective defense system for the body, separating the internal and external environments to prevent the passage of potentially harmful substances [34]. Researchers have found that the closure of the intestinal mucosa requires a complete epithelial structure and an efficient apical cell connection complex [35,36]. However, under the dual influence of HL changing intestinal flora and AP releasing pro-inflammatory factors, intestinal mucosal permeability improves to break the closed state. This phenomenon further stimulates the body to release inflammatory cytokines, which exacerbate the HLAP condition to the level of damage to the intestinal functional barrier. In this study, different doses of RS reduced the levels of serum D-LA, DAO, and ET; increased the content of sIgA; and significantly increased the expression level of mRNA of ZO-1, occludin, CRAMP, and DEFB1 in the intestinal mucosa of rats in each RS group. Related pathological section results also characterize the protective effect of RS on the microstructure of the small intestinal mucosa. In summary, it was proved that RS could up-regulate the mRNA expression levels of the main tight junction proteins and antimicrobial peptides in the intestine, and promote the secretion of immune antibodies, thereby improving the permeability of the intestinal mucosa and maintaining the integrity of the intestinal barrier.

## 4. Materials and Methods

### 4.1. Materials

Purple speckled kidney beans (*Phaseolus vulgaris*) were provided by the reclamation area of Heihe (China). Male Wistar rats (SPF, Permit number: SCXK (Liao) 2020-0001) were purchased from Changsheng Biotechnology Co., Ltd. (Benxi, China). Caerulein (≥99%) was obtained from Med Chem Express (Monmouth Junction, NJ, USA). Octreotide acetate injection was from Novartis Pharmaceutical Co., Ltd. (Beijing, China). Assay kits for tumor necrosis factor-α (TNF-α), interleukine-1 beta (IL-1β), interleukine-6 (IL-6), diamine oxidase (DAO), endotoxin (ET), and D-lactic acid (DLA) were provided by Calvin Biotechnology Co., Ltd. (Suzhou, China). Assay kits for triglyceride (TG), total cholesterol (TC), and myeloperoxidase (MPO) were purchased from the Institute of Bioengineering (Nanjing, China). The NCBI primer designing tool designed RT-PCR amplification primers, and the primer sequence was synthesized by Shenggong Biotechnology Co., Ltd. (Shanghai, China).

### 4.2. Preparation and Purification of Kidney Bean RS

The kidney bean starch was weighed and mixed in an Erlenmeyer flask to a starch suspension with a mass fraction of 20%. The temperature of the ultrasonic-microwave collaborative reactor was set at 40 °C, the microwave power at 300 W, and coordinated processing took place for 20 min. After autoclaving at 121 °C for 30 min, we then added pullulanase at a ratio of 9 ASPU/g dry basis, which was shaken at 55 °C for 10 h, and we deactivated the enzyme by boiling in water for 10 min. It was then refrigerated at 4 °C for 24 h. The aged gelatin starch was dried in an oven at 50 °C for 12 h. It was crushed at high speed through an 80-mesh sieve to obtain kidney bean RS. Excessive thermostable α-amylase and saccharification enzymes were utilized to purify RS.

### 4.3. Structure of RS

The structural characteristics of kidney bean RS were determined using a scanning electron microscope, X-ray diffractometer (Empyrean, PANalytical B.V., Almelo, Overijssel, Netherlands), and laser particle size analyzer (Bettersize2000, Better, China) [37,38,39]. Meanwhile, a GI20 automatic in vitro simulated digestion system (NutriScan GI20, Next Instruments, Condell Park, NSW, Australia) was applied to determine the glycemic index of RS.

### 4.4. Animal Feeding and Administration

Normal male Wistar rats were housed in rat cages for animal experiments and maintained under a simulated ambient environment that was kept at a cycle of illumination for 12 h, a constant temperature (24 ± 2) °C, and a relative humidity (50 ± 5)%. The experimental rats were randomly divided into six groups based on body weight after seven days of adaptive feeding (*n* = 6), including control group (CON), model group (MOD), simvastatin group (SV), and RS groups (H-RS, M-RS, and L-RS). In the initial stage of the experiment, except for the CON rats the other groups of rats were fed a hyperlipidemia diet (Table 4) for 4 weeks to induce a hyperlipidemia model. During this period, it was judged whether the model was successful based on the serum TG and TC concentration of rats (the model was established when the contents of serum TG and TC in each group increased to 2~3 times those of the CON). The acute pancreatitis model was induced by intraperitoneal injection of caerulein (50 μg·kg^−1^) for 8 consecutive times [40,41,42]. The SIM was pre-fed with simvastatin (10 mg·kg^−1^) and injected with octreotide acetate in the tail vein after intraperitoneal injection (10 mg·kg^−1^). RS groups were pre-fed with different doses of RS (5.4 g·kg^−1^, 2.7 g·kg^−1^, 1.35 g·kg^−1^) for 6 weeks. Rats were humanely sacrificed to obtain blood and related issues. All experiments were carried out according to the P.R. China legislation and we strictly followed the international guidelines of the Institutional Animal Care and Use Committee. The rat handling and the experimental protocol were also performed according to the Directive 2010/63/EU on the protection of animals used for scientific purposes (European Parliament and Council, Directive 2010/63/EU of 22 September 2010 on the protection of animals used for scientific purposes. Off. J. Eur. Union 2010, L276, 33–79).

### 4.5. Serum Analysis

Blood was collected from the portal vein and centrifuged at 4000× *g* (4 °C, 20 min). The serum was collected and dispensed by using sterile pipets; one part was used to determine serum lipase and amylase, and the others were stored at −80 °C until analysis. Serum samples were equilibrated at 4 °C for 10 min and then amylase and lipase levels were analyzed using a Vet Test 8008 automatic biochemical analyzer (IDEXX Bioresearch, Westbrook, ME, USA, USA). Results are expressed in international units per liter (U/L). Serum was removed from −80 °C and thawed to analyze related indicators. The expression and activity of TNF-α, IL-1β, IL-6, ET, sIgA, DAO, and DLA were determined according to kit instructions.

### 4.6. Histology Analysis

Portions of fresh, independent pancreatic tissue in each group of rats were randomly collected and weighed. The degree of pancreatic edema was assessed by the ratio of the mass lost from a completely dried pancreas to the wet weight of the pancreas [43]. Pancreatic tissue was formulated into a homogenized medium according to experimental methods and evaluated based on the mechanism by which myeloperoxidase reduces hydrogen peroxide.

Then, the fresh pancreatic and colon tissue were fixed in a volume fraction of 10% formaldehyde solution and dehydrated with gradient ethanol, soaked to transparent xylene, waxed, and embedded to prepare hematoxylin-eosin-stained sections. The pathological changes in pancreatic tissue were observed under a ICC50 light microscope (LEICA, Wetzlar, Germany).

### 4.7. RNA Extraction and Quantitative PCR

RNA was separated using a UNIQ-10 column Trizol total RNA extraction kit (SK1321). The frozen colon tissue was homogenized by a homogenizer at a ratio of 0.5 mL Trizol per 15 to 25 mg of tissue. The homogenate was allowed to stand at room temperature for 5~10 min to separate the nucleoprotein from the nucleic acid completely, and then the total RNA was obtained according to the instructions. The cDNA was synthesized by reverse transcription kit manipulation instructions for reverse transcription. Specific primers were designed based on the gene sequence (Table 5) [43]. β-actin was used as the reference gene for quantitative detection by real-time fluorescent quantitative PCR, and the cycling conditions refer to Piorkowski, with appropriate adjustments as follows: the thermocycler profile was 3 min at 95 °C, followed by 5 s at 95 °C and 30 s at 60 °C for 45 cycles [44]. Finally, the data were analyzed using the 2^−ΔΔCt^ method.

### 4.8. Statistical Analysis

All data are expressed as the mean ± standard error of the mean. Differences between data were analyzed using an independent sample *T*-test and one-way ANOVA. Statistical analyses were conducted using SPSS 20.0 statistical software with *p* < 0.05 considered statistically significant.

## 5. Conclusions

In conclusion, the internal composition and stable internal structure of RS reduced the area of contact between RS and digestive enzymes, and increased its residence time in the intestine. These factors contributed to it performing its physiological functions. In addition, the RS reduced serum lipid content, improved pancreatic tissue edema and inflammatory damage, increased the expression of tight junction proteins, and reduced intestinal mucosal permeability. This study demonstrates that RS has the potential to prevent HLAP, and it will hopefully provide a foundation for the design and development of kidney-bean-based functional foods.

## Figures and Tables

**Table 1:** Mean particle size of kidney bean starch and resistant starch.

Sample	Low Particle Size D10/μm	Median Particle Size D50/μm	High Particle Size D90/μm	Volume Average Particle Size/μm	Specific Surface Area/m^2^·g
Starch	18.65 ± 0.18 ^a^	28.77 ± 0.75 ^a^	40.85 ± 3.54 ^a^	28.88 ± 1.46 ^a^	0.119 ± 0.011 ^a^
RS	41.63 ± 0.22 ^b^	166.30 ± 2.87 ^b^	355.2 ± 2.12 ^b^	184.60 ± 3.39 ^b^	0.044 ± 0.013 ^b^

Values followed by different lower-case letters in the same column are significantly different from each other (*p* < 0.05).

**Table 2:** Dietary intake of RS affected the body weight and pancreas weight of HLAP rats.

Parameter	Model Group (MOD)	Control Group (CON)	Simvastatin Group (SV)	Low-Dose RS Group (L-RS)	Medium-Dose RS Group (M-RS)	High-Dose RS Group (H-RS)
Body mass/g	471.16 ± 8.08 ^f^	378.73 ± 10.15 ^a^	405.88 ± 7.19 ^b^	451.67 ± 6.26 ^e^	429.78 ± 8.98 ^d^	418.35 ± 5.19 ^c^
Pancreas mass/g	1.295 ± 0.054 ^a^	1.789 ± 0.019 ^f^	1.520 ± 0.023 ^c^	1.468 ± 0.024 ^b^	1.666 ± 0.035 ^d^	1.748 ± 0.041 ^e^
Pancreas index/%	0.289 ± 0.042 ^a^	0.455 ± 0.041 ^e^	0.375 ± 0.012 ^c^	0.325 ± 0.010 ^b^	0.388 ± 0.016 ^cd^	0.418 ± 0.014 ^d^

Values followed by different lower-case letters in the same line are significantly different from each other (*p* < 0.05).

**Table 3:** Blood-serum-related parameter levels in each group of rats.

Parameter	MOD	CON	SV	L-RS	M-RS	H-RS
TC (mmol/L)	4.08 ± 0.13 ^e^	0.81 ± 0.14 ^a^	2.44 ± 0.11 ^d^	2.32 ± 0.21 ^d^	1.55 ± 0.17 ^c^	1.25 ± 0.14 ^b^
TG (mmol/L)	2.22 ± 0.12 ^f^	0.67 ± 0.05 ^a^	1.48 ± 0.07 ^c^	1.94 ± 0.13 ^e^	1.74 ± 0.05 ^d^	1.25 ± 0.08 ^b^
AMLY (U/L)	4366.16 ± 117.84 ^f^	1280.27 ± 71.93 ^a^	2660.19 ± 105.55 ^b^	3637.9 ± 61.05 ^e^	3218.61 ± 60.65 ^d^	3010.32 ± 65.86 ^c^
LIPA (U/L)	1016.58 ± 35.73 ^e^	107.97 ± 19.91 ^a^	780.51 ± 20.57 ^d^	793.22 ± 15.39 ^d^	674.44 ± 32.76 ^c^	523.05 ± 27.66 ^b^
TNF-α (pg/mL)	197.60 ± 2.72 ^f^	60.13 ± 4.10 ^a^	110.60 ± 2.37 ^b^	181.67 ± 3.25 ^e^	140.67 ± 4.39 ^d^	130.33 ± 4.29 ^c^
IL-6 (pg/mL)	110.67 ± 4.59 ^d^	12.33 ± 1.53 ^a^	50.67 ± 3.61 ^bc^	90.00 ± 2.00 ^c^	75.33 ± 4.18 ^b^	55.33 ± 2.07 ^b^
IL-1β (pg/mL)	86.88 ± 2.46 ^f^	15.00 ± 1.43 ^a^	53.80 ± 0.82 ^b^	80.00 ± 1.26 ^e^	72.82 ± 1.81 ^d^	67.00 ± 2.03 ^c^
DAO (ng/mL)	95.07 ± 4.21 ^e^	57.58 ± 3.51 ^a^	70.51 ± 1.41 ^b^	85.98 ± 3.32 ^d^	72.13 ± 4.96 ^bc^	75.31 ± 2.31 ^c^
DLA (μmol/L)	38.76 ± 2.27 ^f^	23.57 ± 0.62 ^a^	26.72 ± 1.34 ^b^	36.76 ± 1.05 ^e^	35.08 ± 1.14 ^d^	28.41 ± 0.92 ^c^
ET (EU/mL)	71.84 ± 1.87 ^e^	53.16 ± 2.58 ^a^	57.26 ± 1.82 ^c^	67.74 ± 1.12 ^d^	56.37 ± 1.70 ^bc^	54.84 ± 2.27 ^ab^
sIgA (μg/mL)	19.01 ± 0.73 ^a^	25.04 ± 1.16 ^c^	26.75 ± 1.75 ^d^	19.49 ± 0.78 ^a^	21.28 ± 1.39 ^b^	22.67 ± 1.38 ^b^

Values followed by different lower-case letters in the same line are significantly different from each other (*p* < 0.05).

**Table 4:** The feed composition of rats in each group.

Ingredients (%)	CON	MOD	SV	L-RS	M-RS	H-RS
Corn starch	73.5	53.51	53.51	53.51	53.51	53.51
Wheat bran	20	14.6	14.6	14.6	14.6	14.6
Fish meal	5	3.6	3.6	3.6	3.6	3.6
Farina	1	0.73	0.73	0.73	0.73	0.73
Sodium salt	0.5	0.56	0.56	0.56	0.56	0.56
Cholesterol	/	1.2	1.2	1.2	1.2	1.2
Egg yolk powder	/	5.8	5.8	5.8	5.8	5.8
Sucrose	/	10	10	10	10	10
Lard	/	10	10	10	10	10

**Table 5:** The gene sequence of each primer.

Gene Name	Forward Primer	Temperature/°C	Length/bp
ZO-1	GAGATGAGCGGGCTACCTTA GCTGTGGAGACTGTGTGGAAT	57.2 57.0	210
Occludin	TGGGACAGAGCCTATGGAAC ACCAAGGAAGCGATGAAGC	57.2 57.5	197
CRAMP	TCACTGTCACTGCTATTGCTCCT CCTTCACTCGGAACCTCACAT	59.3 58.9	208
DEFB1	CTGCCCATCTCATACCAAACTAC TTTACAATCCCTTGCTGTCCTT	58.4 58.5	112

## Data Availability

The data presented in this study are available on request from the corresponding author.

## Sample Availability

Samples of the compounds are not available from the first author.
